# Scale-Up of HIV Antiretroviral Therapy and Estimation of Averted Infections and HIV-Related Deaths — Uganda, 2004–2022

**DOI:** 10.15585/mmwr.mm7204a2

**Published:** 2023-01-27

**Authors:** Emilio Dirlikov, Joseph Kamoga, Stella Alamo Talisuna, Jennifer Namusobya, Daniel E. Kasozi, Juliet Akao, Estella Birabwa, Jennifer A. Ward, Bill Elur, Ray W. Shiraishi, Carl Corcoran, Vamsi Vasireddy, Richard Nelson, Lisa J. Nelson, Mary Borgman, Eleanor Namusoke Magongo, Linda Nabitaka Kisaakye, Cordelia Katureebe, Wilford Kirungi, Joshua Musinguzi, PEPFAR Uganda, PEPFAR Uganda, Natalie E. Brown, Daniel Bogere, Jessica T. Conley, Arthur G. Fitzmaurice, Jennifer S. Galbraith, Joseph Kabanda, Geoffrey Kabuye, Julius N. Kalamya, Charles Kavuma, Herbert S. Kiyingi, Deus Lukoye, Stephen Malinzi, Lisa A. Mills, Kenneth Musenge, Diriisa Musisi, Kenneth Mwambi, Christina W. Mwangi, Grace A. Namayanja, Phoebe M. Namukanja, Sophie Nantume, Pamela Nasirumbi Muniina, Esther R. Nazziwa, Thomas Nsibambi, Thomas Nsibambi, Jonathan Ntale, Robert Ochai, Samuel Sendagala, Alfred S. Lutaaya, Hardson K. Tibihenda, Rachel K. Kwezi, Jaffer Byawaka Majugu, Jacqueline Calnan, Immaculate N. Ddumba, Seyoum Dejene, Bikokye W. Kafeero, Garoma Kena, Dalsone Kwarisiima, Sheila Kyobutungi, Haruna Lule, Ismail Mbabali, Norbert Mubiru, Emmanuel Mugisa, Miriam Murungi, Aleathea D. Musah, Suzan K. Nakawunde, Victoria Nakiganda, Jennifer Namusobya, Saidah Nankabirwa, Peter Niwagaba, Esther K. Nkolo, Tamara Nsubuga-Nyombi, Babatunji Odelola

**Affiliations:** ^1^Uganda CDC Country Office, Kampala, Uganda; ^2^U.S. President’s Emergency Plan for AIDS Relief, Department of State, Kampala, Uganda; ^3^United States Agency for International Development, Kampala, Uganda; ^4^U.S. Department of Defense, Kampala, Uganda; ^5^Division for Global HIV and TB, Center for Global Health, CDC; ^6^AIDS Control Program, Uganda Ministry of Health, Kampala, Uganda.; Department of State; CDC; CDC; CDC; CDC; CDC; CDC; CDC; CDC; CDC; CDC; CDC; CDC; CDC; CDC; CDC; CDC; CDC; CDC; CDC; CDC; CDC; CDC; CDC; CDC; CDC; CDC; U.S. Department of Defense; U.S. Department of Defense; President’s Emergency Plan for AIDS Relief; Department of State; U.S. Agency for International Development; U.S. Agency for International Development; U.S. Agency for International Development; U.S. Agency for International Development; U.S. Agency for International Development; U.S. Agency for International Development; U.S. Agency for International Development; U.S. Agency for International Development; U.S. Agency for International Development; U.S. Agency for International Development; U.S. Agency for International Development; U.S. Agency for International Development; U.S. Agency for International Development; U.S. Agency for International Development; U.S. Agency for International Development; U.S. Agency for International Development; U.S. Agency for International Development; U.S. Agency for International Development; U.S. Agency for International Development; U.S. Agency for International Development; U.S. Agency for International Development; U.S. Agency for International Development

On January 28, 2003, the U.S. President’s Emergency Plan for AIDS Relief (PEPFAR), the largest commitment by any nation to address a single disease in history, was announced.* In April 2004, the first person in the world to receive PEPFAR-supported antiretroviral therapy (ART) was a man aged 34 years in Uganda. Effective ART reduces morbidity and mortality among persons with HIV infection (*1*) and prevents both mother-to-child transmission (MTCT) (*2*) and sexual transmission once viral load is suppressed to undetectable levels (<200 viral copies/mL) (*3*). By September 2022, more than 1.3 million persons with HIV infection in Uganda were receiving PEPFAR-supported ART, an increase of approximately 5,000% from September 2004. As indicators of the ART program’s effectiveness, a proxy MTCT rate decreased 77%, from 6.4% in 2010 to 1.5% in 2022, and the viral load suppression rate (<1,000 viral copies/mL) increased 3%, from 91% in 2016 to 94% in September 2022. During 2004–2022, ART scale-up helped avert nearly 500,000 HIV infections, including more than 230,000 infections among HIV-exposed infants, and approximately 600,000 HIV-related deaths. Going forward, efforts will focus on identifying all persons with HIV infection and rapidly linking them to effective ART. PEPFAR remains committed to continued strong partnership with the Government of Uganda, civil society, and other development partners toward sustainable solutions aligned with the Joint United Nations Programme on HIV/AIDS (UNAIDS) fast-track strategy to ending the global AIDS epidemic by 2030^†^ and safeguarding impact achieved in the long term.

Local cases of AIDS were first recognized in Uganda in the early 1980s (*4*). In October 1986, the Uganda AIDS Control Program (ACP) was established within the Ministry of Health, initially focused on HIV prevention and palliative care, because of the lack of treatment options at the time (*5*). HIV prevalence started to decline in the early 1990s, linked to reductions in casual sex and increased protective sexual behavior (e.g., condom use) (*6*,*7*). In 2002, ACP established a national HIV MTCT prevention program (*8*), after the HIVNET 012 trial, which was conducted in Uganda and found that nevirapine could prevent MTCT (*9*).

In April 2004, Uganda was the first country in the world to provide PEPFAR-supported ART. Since then, ART eligibility criteria have expanded from an initial focus on patients with advanced disease (e.g., CD4 count <200 cells/*μ*L). In 2012, “Option B+” expanded ART eligibility to all pregnant and breastfeeding women with HIV infection (*8*), and in 2015, “Treat All” expanded ART eligibility to all persons with HIV infection regardless of disease severity or other criteria.^§^ In 2018, Uganda introduced dolutegravir-based regimens (e.g., tenofovir, lamivudine, and dolutegravir [TLD]), with the goal of improving ART effectiveness.^¶^ Since March 2020, the COVID-19 pandemic has affected medical services, including the HIV program; for example, movement restrictions limited patients’ ability to attend clinics for appointments and ART refills (*10*). In addition to clinical services and commodity procurement, PEPFAR has also supported health system–strengthening activities, including workforce capacity building and support to health workers; leadership and governance capacity building; development of financing, information, laboratory, and supply chain systems; and integration of HIV services into the general health system.** PEPFAR Uganda has worked in collaboration with other stakeholders, including UNAIDS and the Global Fund to Fight AIDS, Tuberculosis, and Malaria, to ensure optimized resource utilization in support of the Government of Uganda. By 2021, UNAIDS estimated that there were 1.4 million (range = 1.3–1.6 million) persons with HIV infection in Uganda, with an estimated 54,000 (range = 43,000–69,000) new infections occurring annually.^††^

To describe the scale-up of PEPFAR-supported ART, PEPFAR Monitoring, Evaluation, and Reporting and archival programmatic data^§§^ were analyzed by fiscal year (October–September); data permitting comparison by sex and age group (adults [persons aged ≥15 years] and children and adolescents [persons aged <15 years]) have been available since 2005. Before October 2018, persons with HIV infection on ART were defined as clients at a PEPFAR-supported site if ≤90 days had elapsed since their last appointment; in October 2018, this definition changed to ≤28 days since the last appointment. The proxy MTCT rate was calculated as the number of HIV-exposed infants during pregnancy or the breastfeeding period (i.e., 18 months postpartum) who had a positive HIV test result among those who received testing (data available for 2010–2022). Viral load suppression was defined as <1,000 HIV viral copies/mL, and the suppression rate was calculated as the number of persons with HIV infection with viral load suppression among those who had received a viral load test (data available for 2016–2022). The 2021 UNAIDS Spectrum AIDS Impact Model (AIM) and Goals ASM models were used to estimate the number of infections averted, including among HIV-exposed infants, and HIV-related deaths averted by mid-year (July–June). Both models use national program statistics, survey and surveillance data, and study-derived epidemiologic parameters to calibrate structured models of HIV transmission and produce indicators such as incidence and mortality.^¶¶^ To estimate the number of infections and deaths averted in the absence of PEPFAR, the number of persons with HIV infection on ART were interpolated using program data from 2003 when an estimated 2% of persons with HIV infection were on ART, with 2% of the program numbers for 2021; the number of women reached by the MTCT prevention program was held constant from 2003 levels. Data from the electronic medical records for the first person to receive PEPFAR-supported ART were abstracted from the clinic where he first received ART and from the clinic where he last received ART. This activity was reviewed by CDC and conducted consistent with applicable federal law and CDC policy.***

During September 30, 2004–September 30, 2022, the number of persons with HIV infection on PEPFAR-supported ART increased 4,884%, from 26,365 to 1,313,952 (Figure 1). During September 30, 2005–September 30, 2022, the number of adults with HIV infection receiving PEPFAR-supported ART increased 2,687%, from 45,061 to 1,255,983, and the number of children with HIV infection on PEPFAR-supported ART increased 1,167%, from 4,577 to 57,969. The number of women and men with HIV infection on PEPFAR-supported ART increased 3,723%, from 28,836 to 853,103, and 2,115%, from 20,802 to 460,849, respectively.

The proxy MTCT rate declined from 6.4% (2,327 HIV-positive infants exposed during pregnancy or 18 months postpartum among 36,119 tested) in 2010 to 1.5% (1,060 of 71,265) in 2022 (Figure 2). During 2016–2022, the number of viral load tests conducted increased 130%, from 526,936 to 1,213,707, and the viral load suppression rate increased from 91% (479,915 of 526,936) to 94% (1,145,839 of 1,213,707) (Figure 2). In September 2022, viral load suppression rates were higher among adults (95% [1,116,888 of 1,179,551]) than among children (85% [28,951 of 34,156]), and slightly higher among women (95% [757,248 of 797,462]) than among men (93% [388,591 of 416,245]).

During 2004–2022, an estimated 491,345 HIV infections were averted, including 231,833 among HIV-exposed infants. Annually, a median of 21,408 HIV infections were averted, ranging from 913 in 2004 to 57,171 in 2022 (Figure 3). During this period, an estimated 586,074 deaths were averted, ranging from 1,138 in 2004 to 48,348 deaths in 2022 (annual median = 32,179).

## Case Report

In February 2004, a Ugandan man aged 34 years received a diagnosis of HIV infection. The results of a CD4 test conducted in March was 1 cell/*μ*L. In April, he became the first person in the world to receive PEPFAR-supported ART, receiving stavudine-lamivudine-nevirapine (D4T-3TC-NVP). In May 2020, he received a course of tuberculosis preventive therapy, which he completed in November 2020. Since March 2021, he has not received a diagnosis of active tuberculosis disease or an HIV-related opportunistic infection. In January 2021, he was transitioned to TLD, and as of September 2022, he receives 4-month multimonth dispensing. His most recent viral load test conducted in March 2022 indicated viral load suppression. As a result, he was eligible for and enrolled in a fast-track drug refill program.

## Discussion

Twenty years after the announcement of PEPFAR, the program’s first patient is now aged 53 years and remains on PEPFAR-supported ART with suppressed viral load. Sustained efforts substantially expanded ART in Uganda (4,884% increase), and as of September 2022, more than 1.3 million persons with HIV infection were receiving PEPFAR-supported ART. During 2020–2022, HIV services adapted to the COVID-19 pandemic (*10*), with an increased number of persons with HIV infection on PEPFAR-supported ART (98,012) and increased viral load suppression rates. Treatment is effective, as indicated by increased viral load suppression rates, especially after the introduction of dolutegravir-based ART; and since 2004, ART scale-up averted approximately 600,000 HIV-related deaths. Treatment is also prevention, as indicated by decreased MTCT rates, and since 2004, ART scale-up has contributed to averting nearly 500,000 estimated infections, including more than 230,000 estimated infections among HIV-exposed infants.

Despite tremendous gains, persons with HIV infection currently not on ART and those without viral load suppression are at risk for poor clinical outcomes and can transmit HIV, potentially leading to new infections. Among adults, viral load suppression rates have increased to 95%. Rates among men are slightly lower than those among women, and additional efforts are needed to ensure that children receive individually optimized ART, given that viral load suppression rates remain <90% among children. Observed differences in viral load suppression rates derived from program data are substantiated by the 2020–2021 Uganda Population-based HIV Impact Assessment (UPHIA), a nationally representative survey among adults.^†††^ Although MTCT rates have declined, infants continue to be born with or acquire HIV during their first months of life, leading to a lifelong need for ART. The 2020–2021 UPHIA also found that 80.9% of persons with HIV infection knew their status, and 96.1% who knew their status were on ART, indicating linkage to treatment is high, although more efforts are needed to improve case finding.

The findings in this report are subject to at least five limitations. First, indicator definitions and the systems to report data have evolved over time, which might have affected data quality despite continual PEPFAR and national data quality assurance activities. Second, persons with HIV infection can access health services at any site, regardless of residence; therefore, some persons might have been counted more than once. This limitation also prevented direct assessment of ART coverage. Third, the proxy MTCT rate could be an underestimate because HIV-exposed infants who did not have testing were not included. Fourth, the model estimated averted HIV infections and HIV-related deaths based on ART; however, other services (e.g., voluntary medical male circumcision) and contextual factors beyond ART scale up might have contributed. In addition, estimates of the number of infections averted could have been underestimated. Finally, it is not possible to quantify the contribution from PEPFAR and other stakeholders (e.g., UNAIDS and the Global Fund) in support of the Government of Uganda to scale up ART, because investments in infrastructure, leadership, and financing (including commodities) have worked synergistically with PEPFAR investments and programming.

During 2004–2022, PEPFAR supported the scale up of ART (4,884% increase), which averted nearly 600,000 HIV-related deaths and 500,000 infections, including 230,000 infections among HIV-exposed infants. Going forward, efforts will focus on identifying all persons with HIV infection, and rapidly linking them to effective ART. PEPFAR remains committed to continued strong partnership with the Government of Uganda, civil society, and development partners toward sustainable solutions aligned with the UNAIDS fast-track strategy to ending the global AIDS epidemic by 2030 and safeguarding impact in the long term.

**Summary**

**What is already known about this topic?**

In January 2003, the U.S. President’s Emergency Plan for AIDS Relief (PEPFAR) was launched. In April 2004, Uganda became the first country to provide PEPFAR-supported antiretroviral therapy (ART).

**What is added by this report?**

During 2004–2022, the number of persons with HIV infection receiving PEPFAR-supported ART increased by almost 5,000%, to more than 1.3 million, averting nearly 500,000 HIV infections, including more than 230,000 among HIV-exposed infants, and approximately 600,000 HIV-related deaths.

**What are the implications for public health practice?**

Going forward, efforts will focus on identifying all persons with HIV infection and linking them to effective ART. PEPFAR remains committed to continued strong partnership with the Government of Uganda and other stakeholders toward ending the global AIDS epidemic by 2030 and safeguarding the long-term impact.

**FIGURE 1:**
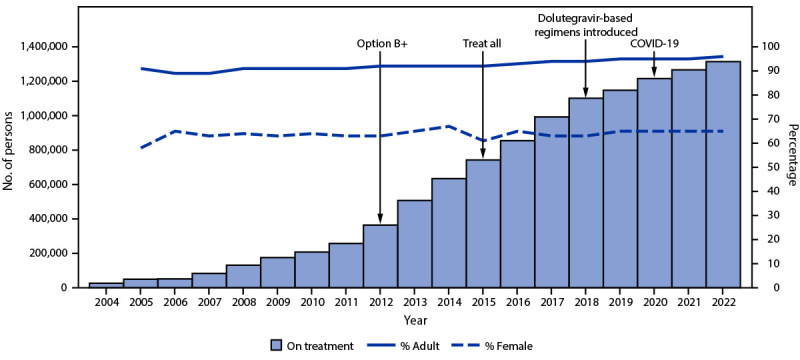
Cumulative number of persons with HIV infection receiving PEPFAR-supported antiretroviral therapy,* with percentage who are adults and who are female^†^ — Uganda, fiscal years 2004–2022^§^ **Abbreviations:** ART = antiretroviral therapy; PEPFAR = President’s Emergency Plan for AIDS Relief. * Before October 2018, persons with HIV infection on ART were defined as clients at a PEPFAR-supported site with ≤90 days since last appointment; in October 2018, this definition changed to ≤28 days since last appointment. “Option B+” expanded ART eligibility to all pregnant and breastfeeding women with HIV. “Treat All” expanded ART eligibility to all persons with HIV infection regardless of disease severity or other criteria. The main dolutegravir-based regimen used in Uganda is tenofovir-lamivudine-dolutegravir. The first case of COVID-19 in Uganda was identified in March 2020. † Data on percentages of age and sex to calculate percentage adults (aged ≥15 years) and female of any age available for 2005–2022. ^§^ October–September. Data represent number of persons with HIV infection on PEPFAR-supported ART on September 30 of each fiscal year.

**FIGURE 2:**
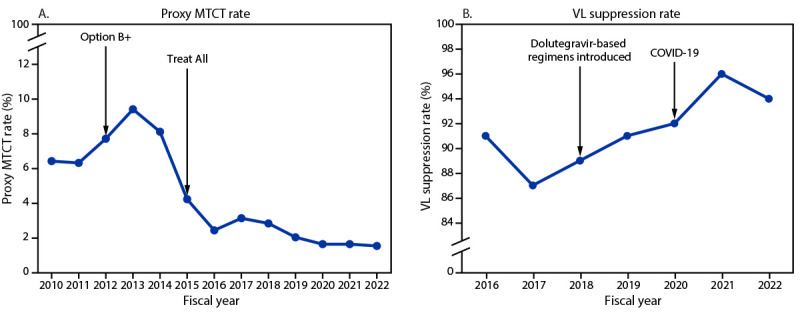
Proxy mother-to-child transmission rate* (A) and viral load suppression rate^†^ (B) reported by PEPFAR-implementing partners — Uganda, fiscal years 2010–2022^§^ **Abbreviations:** ART = antiretroviral therapy; MTCT = mother-to-child transmission; PEPFAR = President’s Emergency Plan for AIDS Relief; VL = viral load. * Number of HIV-exposed infants during pregnancy or the breastfeeding period (i.e., 18 months postpartum) who received a positive HIV test result among those who were tested. Data were available for fiscal years 2010–2022. “Option B+” expanded ART eligibility to all pregnant and breastfeeding women with HIV infection. “Treat All” expanded ART eligibility to all persons with HIV infection regardless of disease severity or other criteria. The main dolutegravir-based regimen used in Uganda is tenofovir-lamivudine-dolutegravir. The first case of COVID-19 in Uganda was identified in March 2020. ^†^ VL suppression defined as <1,000 viral copies/mL; suppression rate calculated as number of persons with HIV infection with VL suppression among those who had a VL test. Data available for fiscal years 2016–2022. ^§^ October 1–September 30. Data represent number of persons with HIV infection on PEPFAR-supported ART on September 30 of each fiscal year.

**FIGURE 3:**
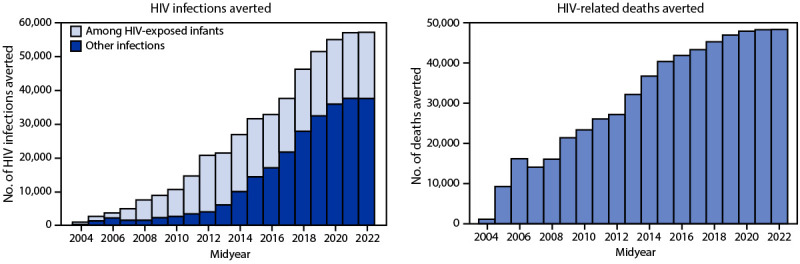
Numbers of HIV infections and deaths averted* — Uganda, mid-years 2004–2022^†^ **Abbreviation:** UNAIDS = United Nations Programme on HIV/AIDS. * Using the 2021 UNAIDS Spectrum AIDS Impact Model and Goals ASM model to estimate the number of infections (including among HIV-exposed infants) and deaths averted. https://www.avenirhealth.org/software-spectrum.php ^† ^Mid-years are July–June.

## Footnotes

*
https://www.state.gov/pepfar/

†
https://www.unaids.org/en/resources/campaigns/World-AIDS-Day-Report-2014

§ https://www.who.int/publications/i/item/treat-all-policy-adoption-and-implementation-status-in-countries

¶
https://www.who.int/news/item/22-07-2019-who-recommends-dolutegravir-as-preferred-hiv-treatment-option-in-all-populations

**
https://nap.nationalacademies.org/catalog/18256/evaluation-of-pepfar

††
https://www.unaids.org/en/regionscountries/countries/uganda

§§ All PEPFAR-supported countries are required to report Monitoring, Evaluation, and Reporting data quarterly. https://www.state.gov/wp-content/uploads/2021/09/FY22-MER-2.6-Indicator-Reference-Guide.pdf

¶¶
https://www.avenirhealth.org/software-spectrum.php

*** 45 C.F.R. part 46.102(l)(2), 21 C.F.R. part 56; 42 U.S.C. Sect. 241(d); 5 U.S.C. Sect. 552a; 44 U.S.C. Sect. 3501 et seq.

†††
https://phia.icap.columbia.edu/uganda-summary-sheet-2020-2021/
